# Psychometric Evaluation of the Abdominal Bloating Social Support Scale

**DOI:** 10.21315/mjms2024.31.4.11

**Published:** 2024-08-27

**Authors:** Nurzulaikha Abdullah, Yee Cheng Kueh, Garry Kuan, Mung Seong Wong, Yeong Yeh Lee

**Affiliations:** 1 Biostatistics and Research Methodology Unit, School of Medical Sciences, Universiti Sains Malaysia, Kelantan, Malaysia; 2 Faculty of Data Science and Computing, Universiti Malaysia Kelantan, Kelantan, Malaysia; 3 Exercise and Sport Science, School of Health Sciences, Universiti Sains Malaysia, Kelantan, Malaysia; 4 Department of Medicine, School of Medical Sciences, Universiti Sains Malaysia, Kelantan, Malaysia; 5 GI & Motility Unit, Hospital Universiti Sains Malaysia, Kelantan, Malaysia

**Keywords:** bloating, social support, adult, development, validation

## Abstract

**Background:**

Abdominal bloating (AB) is a common, bothersome symptom that negatively affects most adults. Although social support may help people suffering from AB, limited validated questionnaire is available. This study aimed to validate the newly developed Abdominal Bloating Social Support (SS-Bloat) scale for the Malaysian context.

**Method:**

We conducted a cross-sectional study in which we used purposive sampling and a self-administered questionnaire. Based on the literature review, experts’ input and in-depth interviews, new items were generated for SS-Bloat scale. Content validity was assessed by experts and pre-tested with 30 individuals with AB. Construct validity was determined based on exploratory factor analysis (EFA) and confirmatory factor analysis (CFA). Reliability was determined based on Cronbach’s alpha and composite reliability (CR).

**Results:**

During the development stage, eight items were generated for SS-Bloat scale and remained the same after content validity and pre-testing. A total of 152 participants with a mean age of 31.27 years old (68.3% female, 32.7% male) completed the questionnaire. Based on the EFA, three problematic items were removed. The total variance explained was 35.6% with acceptable reliability (α = 0.66). The model was then tested using CFA. The initial model did not fit the data well. After several model re-specifications, the final measurement model of SS-Bloat scale fit the data well with acceptable fit indices (comparative fit index [CFI] = 0.994 and Tucker-Lewis index [TLI] = 0.984). The CR was satisfactory with value of 0.84.

**Conclusion:**

SS-Bloat scale was deemed valid and reliable for assessing the level of social support among AB patients. The questionnaire can be useful for both research studies and clinical purposes, as it is easy to use.

## Introduction

Abdominal bloating (AB), one of the most common problems that people face at some point in their lives, affects productivity and well-being (1–5). Although social support can help people deal with AB, there is still a lack of research on social support among people with AB in Malaysia. Psychological factors are important variables to consider in people with AB. Many studies have shown that social support is essential for maintaining physical and psychological health (6, 7). Even though the field of psychiatry has contributed relatively little to developing, testing and implementing effective evidence-based interventions aimed at increasing social support for patients and at-risk populations, there is convincing evidence demonstrating the beneficial effects of social support on medical and psychological well-being (7). Many epidemiological studies have concentrated on further linking measures of social support to physical health outcomes through newer areas, such as support received, provision and relevant pathways, including potential biological (i.e. inflammation) and behavioural (i.e. health behaviour) mechanisms. Interventions attempting to determine the positive effects of social support are also widespread. Although the longer-term effects of such interventions on physical health remain to be determined, such interventions show promise in influencing quality of life in many chronic disease populations (8).

Based on a pilot study, it was found that most of the participants involved in the study (96.1%) wanted to improve their AB symptoms (9). It was reported that AB was a cause for stress by 62.8% and that stress could also cause AB by 46.8%. Poor diet, lack of exercise, stress and an unhealthy lifestyle were reported as causes of AB by 96.2% of the respondents (9). Among the female participants, 50.7% of AB was attributed to menstruation. AB was regarded as the third (out of 14) most important reason to seek medical care, and it was also associated with a decrease in energy levels, food intake and physical functioning (9–11). Thus, proper management of AB is needed to improve healthcare and social support can help. Ioannou et al. (12) proposed that perceived social support was significantly related to lower depressive symptoms, with self-esteem as a mediator. Based on the stress-mobilising hypothesis, stress leads to psychological distress, which encourages individuals to seek social support (13). Furthermore, stress and depression are associated with higher comorbidity. This high comorbidity may explain the relationship between depression and social support (14). The psychological pathways that mediate the association between social support and mental health outcomes need to be further investigated (15), as this could help us understand how to limit the negative effects of low social support on mental health.

In the literature, there are a few available measures of social support in general and specifically for some health issues. Social support questionnaires such as the Duke-UNC Functional Social Support Questionnaire (FSSQ) (16), the Social Support Questionnaire (SSQ) (17), the Social Support Questionnaire Short Form (SSQ6) (18), the Perceived Social Support Questionnaire (F-SozU K-6) (19), the Norbeck Social Support Questionnaire (NSSQ) (20) and the Multidimensional Scale of Perceived Social Support Malay version (MSPSS-M) (21) were used to measure social support for people with a diverse range of population. In this study, the researchers developed a new, specific social support scale for people with AB. This study also aimed to validate psychometric instruments measuring social support among people with AB, specifically in Malaysia. This evidence is necessary to understand the functions of social support and identify areas that need interventions to improve AB symptoms.

## Methods

### Study Design

The study employed a cross-sectional design. All possible participants at the Hospital Universiti Sains Malaysia (HUSM) were approached including visitors or warded patients, accompanying persons, family members, staffs and students. Non-probability sampling method (purposive sampling) was applied when recruiting participants.

The inclusion criteria were a people with AB, aged 18 years old and above, who are cognitively capable of completing the questionnaire and can read, write or understand Malay language, available and ready to take part at the time of data collection and give their consent to participate in the study. To be included in the study, participants would have at least experienced one episode of AB based on answers to verbal questions including “Have you ever experience bloating?” and/or have satisfied the Rome IV criteria for AB. Briefly, the Rome IV criteria (5) for AB are as follow: i) recurrent feeling of AB or visible distention for at least 1 day per week, ii) onset of symptoms at least 6 months before diagnosis, iii) the presence of symptoms for at least 3 months and insufficient criteria to establish other diagnosis and iv) may also co-exist with mild abdominal pain and minor bowel disorders.

Exclusion criteria included presence of any history of organic gastrointestinal (GI) diseases (inflammatory bowel disease, GI infections and colorectal cancer), history of past abdominal surgeries, the current use of drugs which either cause or worsen AB such as opiates and the presence of major psychiatric illnesses such as schizophrenia. Exclusion was performed during screening using questionnaires.

### Instruments

#### Demographic Information

The questionnaire included items related to participants’ demographic characteristics (i.e. age, gender, ethnicity and medical history).

#### Social Support for SS-Bloat Scale

This SS-Bloat scale measures the social support among people with AB with eight items under one subscale (22). The term social support refers to any support given by any individual, either their spouse or closest family member in dealing with AB symptoms either physically or emotionally. It uses 5-point Likert scales format which range from 1 (strongly agree) to 5 (strongly disagree), where higher scores reflect greater social support. Additional one open ended question was included to verify where they receive the social support from either partner, family, friends or others but it was only for reference; no validation was done on this item. It is added to understand who helps the participants in their research.

#### Sample Size

The validity of SS-Bloat scale among people with AB by using exploratory factor analysis (EFA) was examined. The sample size was determined after the new questionnaires were developed. The number of items for the newly developed questionnaire was eight items for SS-Bloat scale. Using the rule of thumb formula, *N* x *p* where *N* is the number of items in each questionnaire and *p* is the constant from 1 to 5. Costello and Corsborne (23) suggest 5 as the minimum *p* per item. Thus, let say *p* = 10 was chosen, the sample size estimated for the questionnaire was 80. However, 100–250 participants were enough for EFA (24, 25). Therefore, the 152 samples used in the EFA stage were still considered acceptable for the present study. Sample size 200 is considered acceptable for CFA according to Myers et al. (26). However, Tabachnick and Fidel (27) argued that sample size of 200 may be too low for complex models with non-normal distributions with missing data. A sample of *n* = 300 cases has also been suggested and graded as good (28, 29). An additional dropout rate of 10% was added into the estimated sample size based on the formula below. The total sample size needed for the study was 330.

### Procedure

The study was conducted in two phases, namely, exploratory and confirmatory.

All possible participants were further screened according to the inclusion and exclusion criteria. Written consent was obtained before inclusion in the study. The present study used the self-reported SS-Bloat scale with additional sociodemographic questions. The participants voluntarily completed the SS-Bloat scale questionnaire and returned it to the researchers. The estimated time to complete the SS-Bloat scale was 10 min–15 min.

For EFA, there were 220 potential participants screened and eventually 205 participants fulfilled the eligibility criteria and were approached to complete the SS-Bloat scale questionnaire. Among all who returned the questionnaires, 152 were complete and usable for the subsequent EFA data analysis. The response rate was 74.1%, which was considered acceptable.

For CFA, there were 355 new potential participants screened and eventually, 330 participants fulfilled the eligibility criteria and were invited to complete the revise version of SS-Bloat scale questionnaire based on EFA. Among all who returned the questionnaires, 323 were complete and usable for the subsequent CFA data analysis. The response rate was 97.9%, which was considered good.

### Data Analysis

**Figure 1:**
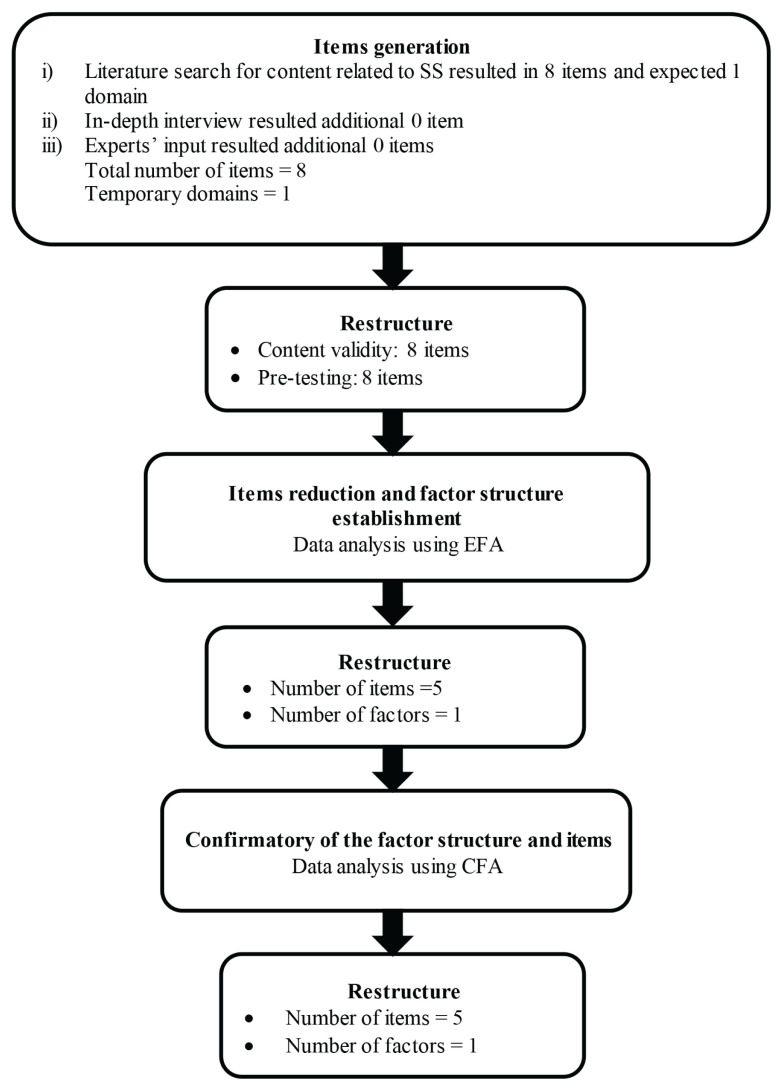
Summary of questionnaire development process for SS-Bloat scale

The new item generation was conducted by the researchers through an extensive literature review related to social support that could encourage improvements in AB symptoms. Based on the literature review, a total of eight items were generated. The research team experts provided no additional related items and supported the one temporary domains from the early draft of the SS-Bloat scale. To cover all the important indicators for the behaviour construct, we conducted an in-depth interview of 12 individuals with AB symptoms. The in-depth interview was conducted using guided questions “Do you receive support from anywhere” with probing questions. The duration of the interview was approximately 30 min. All the recorded interviews were transcribed into a transcript, which was then narratively analysed. Themes were identified from the transcript, a theme list was created and interview segments were coded. Important aspects and critical points from the interviewed individuals were identified. From these interviews, we found no additional item that we added to the SS-Bloat scale’s item pool. Hence, a total of eight items were generated in the initial stage of developing the first draft of the SS-Bloat scale. The responses for each item were rated using a 5-point Likert-scale, from never = 1 to very often = 5. All items were developed in the Malay language, which is the main spoken language in the study’s location. The first draft of the SS-Bloat scale was then examined for its content validity by seven invited experts, who each had at least 10 years of experience in the GI field, psychometric testing, language and questionnaire development. Figure 1 shows the item generation process from the initial stage of development to the final stage of item reduction for the newly developed SS-Bloat scale.

The item content validity index (I-CVI) and scale content validity index (S-CVI) for SS-Bloat scale questionnaire was I-CVI = 0.86–1.00 and S-CVI = 0.98, respectively, which was good (30, 31).

SPSS Statistics software version 26.0 was used to conduct EFA. Factor extraction was conducted using principal axis factoring (PAF). The value of Kaiser-Meyer-Olkin (KMO) test for sampling adequacy and Bartlet’s test of sphericity *P*-value was noted. The Promax rotation method was used as the *P*-value of Bartlett’s test of sphericity of < 0.05 indicates a correlation among items. The number of factors was determined using Kaiser’s eigenvalue where only constructs with eigenvalues of more than 1 should be retained for interpretation (32). The eigenvalue can be interpreted as the total amount of information in a factor. The scree plot (24, 25, 33) was used to determine the final substantial decline in the plot (elbow). The number of dots above the elbow of the plot is considered as the number of factors to be extracted.

Cronbach’s alpha coefficient was used to verify the internal consistency of the items for the SS-Bloat scale where a generally recommended threshold value of 0.60 (34).

CFA was used after the EFA phase to confirm the measurement validity and reliability of the model. As multivariate normality shows non-normal distribution, therefore, maximum likelihood with robust standard errors (MLR) method was used (35). Overall model fitness was inspected using several fit indices such as comparative fit index (CFI) and Tucker-Lewis index (TLI) with a cut-off value of > 0.95, root mean square error of approximation (RMSEA) with a cut-off value of ≤ 0.07, close-fit (ClfitRMSEA) value of > 0.05 and standardised root mean square residual (SRMR) with a cut-off value of ≤ 0.08 (36).

Composite reliability was obtained based on the final CFA model of the SS-Bloat scale. The recommended value for composite reliability is > 0.7 (37) which indicates that a positive convergent validity was achieved and that the items belong to the same factor and share a high proportion of variance.

## Results

### Participants

For the EFA, 152 participants were involved; the mean age was 31.7 years old (SD = 14.36) and 68.3% were female. For the CFA, there were 323 participants; the mean age was 27.69 years old (SD = 11.50) and 59.4% were male. The mean BMI was 24.90 (SD = 14.20), similar between EFA and CFA. The results are summarised in Table 1.

### EFA and Internal Consistency

**Figure 2:**
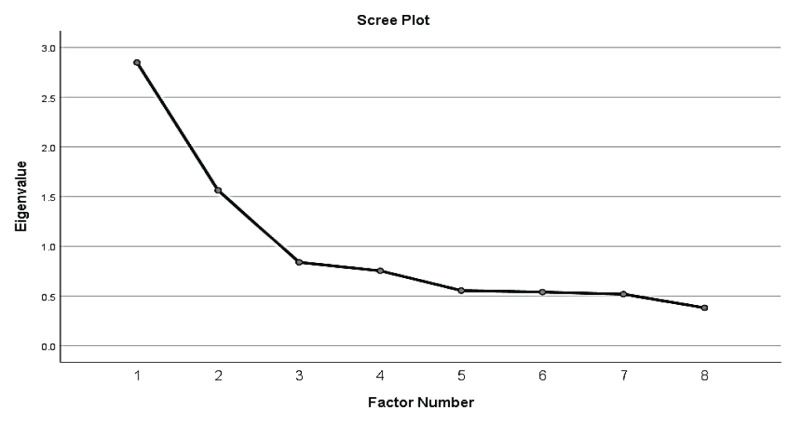
Scree plot for social support construct

The initial principal axis factor analysis of all eight items in SS-Bloat scale indicated sampling adequacy, thus providing a reliable estimate for the current model. The Kaiser-Meyer-Olkin (KMO) test yielded a sampling adequacy of 0.742, which was considered good and the result of the Bartlett’s test of sphericity was significant (*P* < 0.001), again supporting the validity of the EFA model. The items were run with the EFA to explore the domains and two domains with a total variance of 55.14% were found. However, the item combination was considered theoretically inappropriate after examining the factors and their loaded items. The next step was to fix the number of factors to one. The one factor appeared to have an eigenvalue above 1, which indicates acceptable significance. The scree plot is shown in Figure 2. The variance explained by the extracted factors was 35.61%.

Several EFAs were performed sequentially and some items were deleted until all item factor loadings were above 0.40, with no cross-loadings. Using this technique, three items were eventually deleted and five items in one domain (factor) were retained.

Table 2 summarises the results of the EFA and the item factor loadings. The extracted factor represented 35.61% of the variance in the five items. Although there was one item with a factor loading of less than 0.40, it was kept for further analysis, as it was one of the important items for the domain.

The internal consistency was adequate, with a Cronbach’s alpha of 0.66 (Table 3). No items were marked for deletion.

As shown in Table 4, the results of the initial 5-item CFA revealed that CFI and TLI did not achieve the acceptable threshold values. To improve the fit indices, the values and standardised residuals of the modification indices (MIs) were inspected. Model re-specification was done after discussion with other researchers. In the process of model re-specification, a correlation between item residuals was added. Finally, the model fit indices of the modified model were generally acceptable: CFI = 0.994, TLI = 0.984, SRMR = 0.020 and RMSEA (90% CI) = 0.042 (0.000, 0.102) (Table 4).

As Table 5 illustrates, all standardised factor loadings for SS-Bloat scale exceeded the threshold of 0.40 and the average variance extracted (AVE) of each construct was more than 0.61, which indicated good convergent validity. The composite reliability (CR) of the construct was 0.84, which was greater than 0.70.

## Discussion

Developing and validating SS-Bloat scale in the Malay language could be beneficial as a social support tool for use among Malay-speaking populations in South-East Asia, such as Malaysia, Indonesia and the Philippines. The questionnaire was adapted from a few published social support scales and modified to suit AB patients in Malaysia. Although it was important to start with a large number of items so that we would still have enough items to proceed after the EFA reduced the number of items, we believed that with strong and significant items, the scale would capture the content well and that only a few items would be removed. Three items were removed following the EFA. When we checked the questionnaire’s content, the problematic items were redundant and less significant in the scale. The final scale is short, easy to use and concludes social support well. This scale can be considered a short version of a questionnaire measuring social support. However, in future studies, the scale may be modified and validated again for consistency and to suit the future condition.

As previously mentioned, the original version of SS-Bloat scale comprises eight items adapted from other questionnaires and new item generation. All the items were rated using a 5-point Likert scale and thus subjected to EFA to assess the measurement validity of the model. The number of factors extracted was determined based on the screen plot and eigenvalue. The results of the factor loadings were examined and only one item was found to be slightly < 0.3 (item SS5: 0.346). The item was not omitted because it was considered important for measuring social support, especially among people with AB. Asking others for help is one of the ways people seek mental or physical support (38). The final EFA model confirmed that the model is a one-factor model consisting of five items. Additionally, SS-Bloat scale displayed positive internal consistency with an acceptable Cronbach’s alpha of 0.66, which indicated a good level of internal consistency. Hence, this allowed the study to proceed to the CFA phase.

CFA, using the MLR estimator, was performed to confirm whether the model with the five items fit the data well. The data were complete and non-normal, so it suited the Maximum Likelihood Estimate for robust and non-independence (MLR) conditions. MLR was suitable only for data without missing values or incomplete either Missing Completely at Random (MCAR) or Missing at Random (MAR) type. MLR is robust to models in which the data violate the assumption of multivariate normality (39). As the non-normality characteristics were reported from multivariate normality checking and all missing data had been deleted, it was then decided that MLR could be used as the estimator instead of MLM, which can deal with missing data. Overall, model fitness was examined using a few fit indices, as there was no fixed/pre-determined number of fit indices suggested for this purpose. The results of the analysis indicate that SS-Bloat scale was confirmed with one factor and five items. CR was computed after obtaining the final model and demonstrated positive reliability (0.84).

There are no validated measures that can assess psychosocial and psychological variables among people with AB. However, it is important for health practitioners to improve their understanding of the psychological impact of AB, such as social support, which is usually not answerable by just the usual medical examination in order to suggest appropriate treatment for AB. Thus, it is vital to prepare appropriate measurement tools to assess social support for people with AB symptoms. This will help clinicians, patients, researchers and health providers to further understand the manifestation of AB and the need for social support among people with AB.

To date, there are few questionnaires that measure social support in the research field, either for general or specific purposes. However, in this study, the researchers developed a new social support scale that suits the target population, namely people with AB. The newly developed SS-Bloat scale was found to be valid and reliable. The internal consistency using Cronbach’s alpha shows an acceptable result (α = 0.66) (40). In the CFA, all fit indices—including CFI, TLI, SRMR, RMSEA, factor loadings, AVE and CR—showed good validity and fit models with all values above the recommended threshold. This is similar to the short version 12-item Brief 2-Way SSS questionnaire that measures social support promoting older adult well-being with a good Cronbach’s alpha of 0.88 for both domains and all fit indices were above the acceptable value (41). The 11-item Duke Social Support Index (DSSI) also showed good internal consistency (α = 0.71) (42). The Functional Social Support Questionnaire (FSSQ), which was adapted from the Duke-UNC FSSQ, showed good validity (CFI = 0.97, TLI = 0.95, RMSEA 0.07) and reliability (α_total_ = 0.93, α_domain_ = 0.80–0.90) (43). Among the social support questionnaires, SS-Bloat scale is the only one for people with bloating and is the shortest. Therefore, it can be used in future studies to measure social support for people with AB.

This study had a few limitations. SS-Bloat scale was designed to be applied only to the adult population and thus cannot be used to assess adolescents or children. Additionally, the study was conducted in only one centre. Even so, the centre HUSM is a referral centre for Kelantan state and a major hospital in Malaysia. While the target population in this study was patients in hospitals who had experienced AB, due to time and resource limitations, the questionnaires were distributed to all individuals who had experienced AB, including relatives and hospital staff. Participants were selected using multiple methods, including verbal questions, pictogram and the ROME IV items, which is an alternate method of thoroughly diagnosing AB. Since purposive sampling was used and only individuals from the north-eastern region of Peninsular Malaysia were recruited, the study findings may not be generalisable to other regions. Lastly, the results were based on patient-reported outcomes, and although the participants were repeatedly reminded to be as honest and accurate as possible to ensure the accuracy of the results, their responses were prone to response bias.

## Conclusion

This study provided a valid and reliable instrument for measuring social support for people with AB in the Malay language. The final CFA model indicated a good fit to the data, with a valid and reliable model structure. We recommend that SS-Bloat scale be used in future research related to AB and be adapted to other diseases or symptoms.

## Figures and Tables

**Table 1:** Sociodemographic data of participants for EFA (*n* = 152) and CFA (*n* = 323)

Variables	EFA frequency (%)	CFA frequency (%)
Age, years old; mean (SD)	27.69 (11.50)	31.27 (14.36)
Weight, kg; mean (SD)	62.09 (13.36)	62.42 (12.63)
Height, cm; mean (SD)	160.48 (11.79)	158.90 (7.08)
BMI, kg/m^2^; mean (SD)	24.90 (14.20)	24.79 (4.52)
Sex		
Male	192 (59.4)	35 (23.0)
Female	114 (35.3)	97 (63.8)
No response	17 (5.3)	20 (13.2)
Other symptoms		
No	264 (81.7)	93 (61.2)
Yes	49 (15.2)	31 (20.4)
No response	10 (3.1)	28 (18.4)

Note: *n* = frequency;

* mean (SD); Other symptoms = include related symptoms like headache, nausea and abdominal pain

**Table 2:** Factor loading from EFA results (*n* = 151)

No. abbreviated item content	Factor loading
SS1: Ada seseorang yang boleh saya jumpa untuk nasihat dalam menguruskan masalah saya. (There is someone I can turn to for advice about handling my problems.)	–
SS2: Ada seseorang yang menyarankan saya cara untuk menghadapi kembung perut. (There is someone who suggests me ways to deal with bloating.)	0.473
SS3: Saya ada orang yang ambil berat tentang apa-apa yang berlaku terhadap saya (I have people who care about what happens to me.)	0.604
SS4: Saya mendapat kasih sayang dan perhatian. (I get love and care.)	0.669
SS5: Saya ada seseorang yang ikut bersenam bersama. (I have someone who join me exercise together.)	0.346
SS7: Saya mendapat pertolongan apabila saya sakit. (I get help when I am sick.)	0.544
SS8: Saya mempunyai seseorang yang menyarankan saya berjumpa doktor. (I get someone who encouraged me to meet physician.)	–

Note: - = items were removed

**Table 3:** Internal consistency by Cronbach’s alpha for SS-Bloat scale (*n* = 151)

Factor	Corrected item total correlation	Squared multiple correlation	Cronbach’s alpha if item deleted	Cronbach’s alpha
SS2	0.36	0.24	0.62	0.66
SS3	0.47	0.28	0.57	
SS4	0.52	0.28	0.55	
SS5	0.25	0.20	0.66	
SS7	0.44	0.21	0.59	

CFA and Composite Reliability

**Table 4:** Summary for SS-Bloat scale model fit indices (*n* = 323)

CFA model	RMSEA (90% CI)	CFI	TLI	SRMR
Model-1	0.088 (0.046, 0.135)	0.965	0.930	0.034
Model-2^a^	0.042 (0.000, 0.102)	0.994	0.984	0.020

Note:

a Model-2 finalised after removing SS6 and adding correlated items residual; SS2 with SS1

**Table 5:** Standardised factor loading, CR and AVE of SS-Bloat scale measurement model (*n* = 323)

Constructs/items	λ	Model 1	
		AVE	CR
Social Support		0.61	0.84
SS2	0.72		
SS3	0.55		
SS4	0.86		
SS5	0.84		
SS7	0.68		
